# Arthroscopic-assisted uni-portal non-coaxial endoscopic surgery for recurrent osteoid osteoma of the femoral neck: A case report

**DOI:** 10.1097/MD.0000000000044254

**Published:** 2026-01-16

**Authors:** Hongcai Teng, Mingxiu Yang, Danting Xiao, Kai Luo, Wei Dai, Shangyu Liu, Jianming Hu, Jingxin Deng, Haiyi Quan, Yangjie Cai, Liang Mai, Wenxian Huang, En Song, Yun Liu

**Affiliations:** a Department of Spine and Osteopathic Surgery, The First Affiliated Hospital of Guangxi Medical University, Nanning, Guangxi, China; b Department of Traumatic Orthopedic and Hand Surgery, The First Affiliated Hospital of Guangxi Medical University, Nanning, Guangxi, China; c Department of Spine Surgery, Guigang Integrated Traditional Chinese and Western Medicine Orthopedics Hospital, Guigang, Guangxi, China; d Department of Sports Medicine, The First Affiliated Hospital of Kunming Medical University, Kunming, Yunnan, China.

**Keywords:** arthroscopic-assisted uni-portal non-coaxial endoscopic surgery, arthroscopy, AUNES, case report, recurrent osteoid osteoma

## Abstract

**Rationale:**

Osteoid osteoma (OO) is a benign bone tumor predominantly affecting children and adolescents, commonly occurring in the diaphysis of long bones. Traditional open surgery causes damage to surrounding tissues, while radiofrequency ablation requires multiple fluoroscopic sessions, both of which may subject patients to unnecessary harm. However, when OO involves special anatomical structures such as the femoral neck, the difficulty of treatment is further increased. Arthroscopic-assisted uni-portal non-coaxial endoscopic surgery (AUNES), as a novel minimally invasive technique, integrates the advantages of both arthroscopic and endoscopic technologies. It enables precise lesion resection while minimizing tissue damage to the greatest extent. Therefore, this article explores the application value of this technique in femoral neck OO through recurrent cases.

**Patient concerns:**

A 10-year-old female presented with recurrent right thigh pain 11 months after initial open resection of femoral OO, characterized by nocturnal pain responsive to nonsteroidal anti-inflammatory drugs but with no signs of inflammation.

**Diagnoses:**

Imaging studies confirmed recurrent OO at the right femoral neck.

**Interventions:**

The patient underwent AUNES with single-portal tumor resection and allogeneic bone grafting under arthroscopic guidance.

**Outcomes:**

Complete pain resolution was achieved with no recurrence at 1-month follow-up. The technique demonstrated advantages including minimal incision (4 cm), low radiation exposure and early functional recovery.

**Lessons:**

AUNES is a promising treatment for recurrent femoral neck OO, particularly in pediatric patients. While this case shows excellent short-term outcomes, long-term follow-up and larger studies are needed to validate efficacy.

## 1. Introduction

Osteoid osteoma (OO), accounting for approximately 3% of primary bone tumors, is a benign osteogenic neoplasm predominantly affecting children and adolescents aged 5 to 25 years.^[1]^ While most frequently involving the diaphysis of long bones with >50% cases occurring in the femur and tibia, OO rarely occurs in atypical locations such as the skull, scapula, pelvis, ribs, mandible or patella.^[2]^ The characteristic clinical presentation includes localized pain exacerbated at night and typically responsive to nonsteroidal anti-inflammatory drugs. However, conservative management is seldom adopted due to the requirement for prolonged pharmacotherapy and unpredictable clinical outcomes.^[1]^ Traditional open surgical excision, though effective, carries risks of iatrogenic damage to surrounding musculature and soft tissues, resulting in extended recovery periods.

Recent advances in minimally invasive techniques have introduced radiofrequency ablation (RFA) and endoscopic approaches as alternative treatments. While RFA achieves tumor destruction through precise thermal effects, it often requires repeated fluoroscopic guidance for accurate nidus localization, leading to significant radiation exposure.^[3]^ Moreover, the procedure poses challenges in pediatric populations due to lower thermal injury tolerance and difficulties in temperature control during ablation. The management becomes particularly complex when OO involves the femoral neck given its unique anatomical constraints.

To address these limitations, we developed an arthroscopic-assisted uni-portal non-coaxial endoscopic surgery (AUNES) approach for femoral neck OO. This case report retrospectively analyzes the application of AUNES in treating recurrent femoral neck OO.

## 2. Case presentation

### 2.1. Case description

This study was conducted in accordance with the Declaration of Helsinki and approved by the Ethics Committee of the First Affiliated Hospital of Guangxi Medical University (Approval No. 2025-K0198). Written informed consent was obtained from parents of the minor participant included in this research.

The patient was a 10-year-old girl admitted with a chief complaint of “11 months after right femoral tumor resection, recurrent right thigh pain for over 1 month.” Eleven months prior, she had undergone open resection of a right femoral tumor with bone grafting and fusion due to persistent right hip pain for over 6 months. Postoperatively, the incision healed well, hip pain resolved completely and lower limb motorand sensory function remained normal. Histopathology confirmed OO.

**Figure 1.**
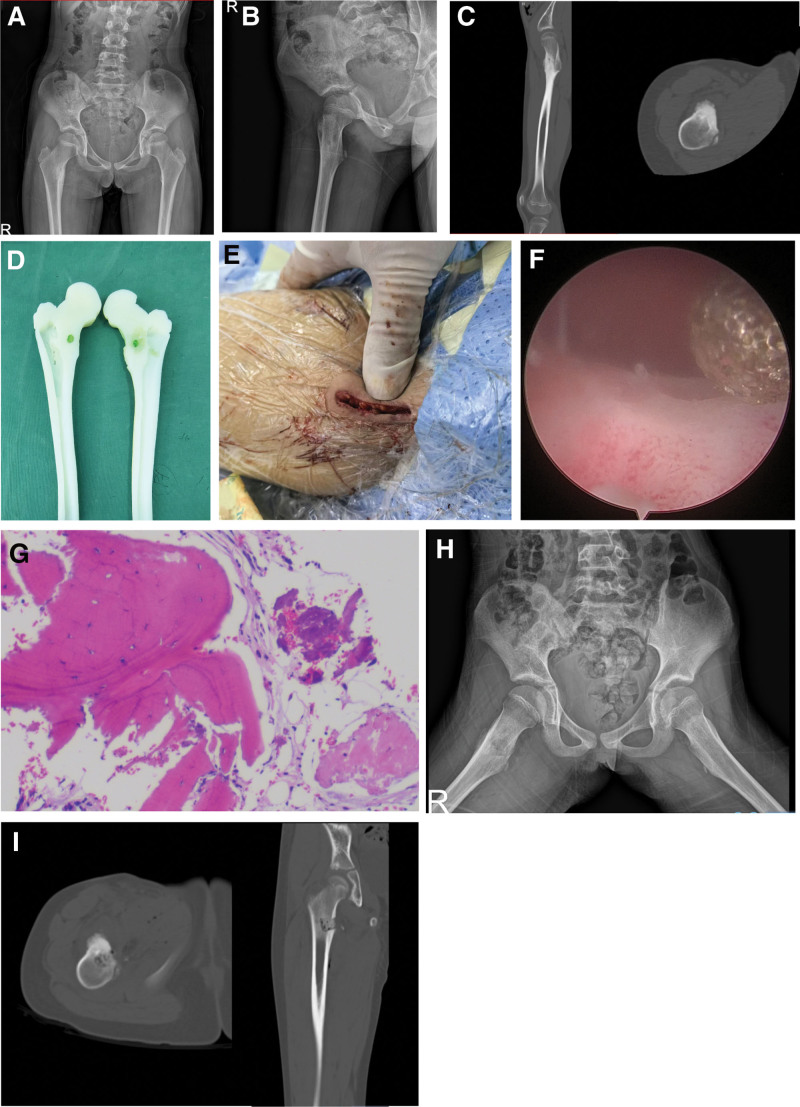
(A) Preoperative pelvic radiograph; (B) right hip lateral radiograph; (C) preoperative CT scans (axial and coronal views); (D) 3D-printed prosthesis model with green markers; (E) intraoperative endoscopic imaging; (F) gross appearance of the surgical incision; (G) HE-stained section of the lesion; (H) postoperative frog-leg radiograph; (I) postoperative CT scan. CT = computed tomography.

One month before admission, she developed recurrent right thigh pain – intermittent stabbing pain without activity-related aggravation but with nocturnal exacerbation. Symptoms improved with nonsteroidal anti-inflammatory drugs or local massage. No swelling, erythema, warmth or fluctuance was observed. At that time, the patient’s visual analogue scale (VAS) was 7 points. Physical examination revealed pain during right hip internal rotation and abduction with limited range of motion, no swelling in the right thigh and no palpable masses (bilateral limb circumference: 30 cm). Both lower extremities demonstrated normal muscle strength and sensation, with intact physiological reflexes and absence of pathological reflexes. She was diagnosed with recurrent OO in the right femoral neck. Imaging studies showed: Pelvic radiograph and right hip lateral radiograph (Fig. 1A, B): postoperative changes following right femoral benign tumor resection with bone graft fusion; suspected bilateral sacroiliitis; and lumbarization of S1; computed tomography (CT) scan (Fig. 1C): postoperative changes at the right lesser trochanter following OO resection; Preoperative 3D-printed prosthesis was prepared with green markers indicating the lesion site (Fig. 1D). The patient subsequently underwent arthroscopic-assisted tumor resection and bone reconstruction on October 23, 2024.

### 2.2. Arthroscopic-assisted tumor resection and bone reconstruction

The patient was placed in the supine position. After standard disinfection and draping of the surgical field, a 4.0 cm longitudinal incision was made on the medial aspect of the right hip (Fig. 1E). The skin and subcutaneous tissues were dissected layer by layer, with careful identification and protection of surrounding neurovascular structures. The interval between the adductor longus and adductor magnus was developed, followed by partial release of the iliacus insertion. Blunt dissection was performed to expose the anterior cortex of the lesser trochanter, where thickened bone without periosteal reaction or soft tissue swelling was observed (Fig. 1F). Adhesions in the surrounding tissues were identified and released, resulting in improved hip joint mobility upon intraoperative manipulation. Intraoperative C-arm positioning was performed only once to confirm correspondence between the surgical site and the preoperative CT-identified lesion location. The endoscopic working cannula and endoscopic system were inserted, connected to the display monitor, initiating direct endoscopic visualization. Radiofrequency ablation electrodes (Bonss’s electrosurgical knife [Jiangsu Bonss Medical Technology Co., Ltd. Models 313 & 302]) were utilized to dissect surrounding soft tissues and achieve hemostasis. Under endoscopic guidance, curettage was performed at the localized site on the anterolateral cortex of the right femoral lesser trochanter using a high-speed drill (Guizhou Zirui Technology Co., Ltd.). With endoscopic magnification and radiofrequency electrodes, extended circumferential curettage around the lesion was conducted and specimens were sent for pathological examination. The surgical site was copiously irrigated with normal saline and carboxymethylcellulose solution to minimize postoperative infection and adhesion formation. The resultant cavity was filled with allogeneic bone graft under compression. After achieving hemostasis, a drainage tube was placed and the wound was closed in layers. The total operative time was 155 minutes, with an estimated blood loss of 20 mL.

### 2.3. Postoperative clinical course

On postoperative day 1, the drainage output was below 50 mL with no significant wound exudate, prompting drain removal. By postoperative day 3, the patient was transferred to rehabilitation for functional exercises. Histopathological examination confirmed tumor-like lesions (Fig. 1G). Follow-up frog-leg radiographs and CT scans demonstrated typical post-resection changes without evidence of right hip dislocation (Fig. 1H, I). The patient’s VAS was 2 points at 1 week postoperatively. At the 1-month follow-up, the incision had healed completely without pain, paresthesia or other complaints. Neurological examination demonstrated normal muscle tone and grade V strength in all extremities.

## 3. Discussion

With advancements in medical technology, the treatment options for OO have expanded significantly, including pharmacotherapy, surgical resection, percutaneous CT-guided RFA and magnetic resonance imaging-guided focused ultrasound surgery.^[4,5]^ In surgical management, different approaches vary not only in the extent of tissue disruption but also in their impact on functional recovery, particularly in pediatric patients where growth considerations are critical, such as open surgery, CT-guided surgical excision and arthroscopic-assisted resection.

In this case, the patient developed recurrent right hip pain with nocturnal exacerbation and responsive nonsteroidal anti-inflammatory drugs symptoms but no signs of inflammation (e.g., erythema, warmth or swelling) following previous right femoral tumor resection and bone graft fusion, suggesting recurrent OO at the femoral neck. Studies have reported a local recurrence rate of 1.7% for surgical treatment of spinal OO, compared to 12.5% for percutaneous CT-guided radiofrequency ablation.^[6]^ The key focus of this patient’s treatment was selecting an appropriate therapeutic strategy for recurrent OO at the femoral neck following previous surgery. For recurrent OO, choosing the optimal treatment approach is critical. It must completely eradicate the lesion to minimize recurrence risk while avoiding secondary tissue damage to preserve normal physiological function. Currently, there are no established treatment guidelines for recurrent OO.^[7]^

If traditional open surgery were selected, complete tumor resection could be achieved. However, given the unique anatomical complexity of the femoral neck, open procedures would cause significant trauma, carry high fracture risks and potentially hinder the pediatric patient’s return to normal daily activities due to prolonged recovery. Percutaneous CT-guided curettage and drilling requires large-bore instruments for operation and cannot guarantee complete tumor resection, making it unsuitable for recurrent cases.^[8]^ Opting for CT-guided RFA would entail excessive radiation exposure, particularly concerning for this female pediatric patient. Currently, arthroscopic OO treatment predominantly employs the conventional dual-portal technique (observation and working portals),^[9,10]^ which presents challenges including a steep learning curve, technical difficulty in maintaining instrument/visual field stability and considerable iatrogenic soft tissue damage from repeated manipulation. These limitations underscore the need for developing approaches with reduced technical demands and minimized invasiveness.

AUNES, a technique initially developed for spinal procedures where it is also termed Arthroscopic-assisted Uni-portal Spinal Surgery (AUSS), integrates the advantages of arthroscopy and spinal endoscopy to enable precise lesion targeting through a single minimally invasive portal with real-time high-resolution visualization, thereby improving surgical accuracy while minimizing peripheral tissue damage. While primarily used for cervical, thoracicand lumbar pathologies, its application in OO remains limited.^[11]^ Unlike traditional RFA requiring repeated fluoroscopic guidance, AUNES utilizes real-time endoscopic visualization to significantly reduce radiation exposure, which is particularly crucial for pediatric patients. Compared to conventional dual-portal endoscopy, AUNES’s single-portal approach combines the instrument and observation channels within 1 flexible incision, eliminating an additional access point and reducing soft tissue trauma.

After comprehensive evaluation of the patient’s condition, we opted for the AUNES approach for tumor resection and bone reconstruction to further minimize the trauma associated with conventional dual-portal arthroscopy. The study by Efthymiadis et al demonstrated that arthroscopic resection of OO achieved a 98% success rate, showing no statistically significant difference compared to percutaneous CT-guided RFA, while being significantly more effective than CT-guided percutaneous curettage and drilling.^[12]^

The selection of surgical approach is a critical determinant of intraoperative success. The single-portal technique eliminates the need to coordinate separate observation and working channels, thereby expanding potential approach options. Compared to conventional anterolateral or anterior approaches, our medial right hip approach provided direct access to the medially located femoral neck lesion while avoiding primary weight-bearing zones of the hip joint. This strategic approach facilitated precise OO localization and excision while maximizing preservation of articular cartilage and healthy bone stock.

To the best of our knowledge, this represents the first documented case utilizing AUNES for recurrent femoral neck OO. Based on this case, we propose that AUNES with bone reconstruction constitutes an effective treatment strategy for femoral neck OO, offering distinct advantages including small incision size, high success rate, negligible radiation exposure and accelerated postoperative recovery.

However, this study has several limitations. First, the reported case involves a single-patient design with a relatively short follow-up period. During the actual surgical procedure, the incision remained larger than ideal due to the surgeon’s limited experience with this specific condition. Postoperative review suggested that the incision size could be further reduced as the surgeon’s proficiency improves, thereby minimizing patient trauma. Additionally, the use of a 30-degree arthroscope could enhance the surgical field of view, allowing for more complete removal of lesions in obscure corner areas. Finally, the endoscopic technique requires the surgeon to have a thorough understanding of the regional anatomy and to perform preoperative surgical simulations to avoid intraoperative damage to critical surrounding structures such as nerves and blood vessels.

## 4. Conclusion

In summary, AUNES represents an effective therapeutic approach for OO. Future multicenter studies with larger cohorts and longer follow-up periods are warranted to further validate these preliminary findings and establish standardized protocols.

## Acknowledgments

The authors would like to thank the patient and her parents for agreeing to publication of this report.

## Author contributions

**Conceptualization:** Hongcai Teng, En Song, Yun Liu.

**Data curation:** Mingxiu Yang, Danting Xiao, Shangyu Liu, Wenxian Huang.

**Investigation:** Jianming Hu, Jingxin Deng.

**Visualization:** Wei Dai, Haiyi Quan.

**Writing – original draft:** Hongcai Teng, Kai Luo, Yangjie Cai, Liang Mai.

**Writing – review & editing:** En Song, Yun Liu.

## References

[1] Napora J Wałejko S Mazurek T. Osteoid osteoma, a diagnostic problem: a series of atypical and mimicking presentations and review of the recent literature. J Clin Med. 2023;12:2721. 37048803 10.3390/jcm12072721 PMC10095250

[2] Carneiro BC Da Cruz IAN Ormond Filho AG. Osteoid osteoma: the great mimicker. Insights Imaging. 2021;12:32. 33683492 10.1186/s13244-021-00978-8 PMC7940467

[3] Fiore F Somma F D’Angelo R Tarotto L Stoia V. Cone beam computed tomography (CBCT) guidance is helpful in reducing dose exposure to pediatric patients undergoing radiofrequency ablation of osteoid osteoma. Radiol Med. 2022;127:183–90. 34958441 10.1007/s11547-021-01439-4 PMC8837556

[4] Bhambhu V Patel PG Mehendiratta D Dalvie S. Complete surgical excision with pre-operative localization of lesion under CT-guidance of osteoid osteoma of the sacrum - a case report. J Orthop Case Rep. 2020;10:56–60. 34169018 10.13107/jocr.2020.v10.i09.1902 PMC8046460

[5] Dai L Zhang X Mei Y. Arthroscopic excision of intra-articular osteoid osteoma of the hip: a case series. Arthroscopy. 2021;37:3104–12. 33865934 10.1016/j.arthro.2021.03.060

[6] Pipola V Tedesco G Spinnato P. Surgery versus radiofrequency ablation in the management of spinal osteoid osteomas: a spine oncology referral center comparison analysis of 138 cases. World Neurosurg. 2021;145:e298–304. 33068800 10.1016/j.wneu.2020.10.050

[7] Shu M Ke J. The surgical management of osteoid osteoma: a systematic review. Front Oncol. 2022;12:935640. 35936708 10.3389/fonc.2022.935640 PMC9355277

[8] Raux S Abelin-Genevois K Canterino I Chotel F Kohler R. Osteoid osteoma of the proximal femur: treatment by percutaneous bone resection and drilling (PBRD). A report of 44 cases. Orthop Traumatol Surg Res. 2014;100:641–5. 25217029 10.1016/j.otsr.2014.05.017

[9] He Y Li X Tu ZX. Arthroscopic treatment of osteoid osteoma in the posterior proximal tibia: a case report and literature review. Medicine (Baltimore). 2024;103:e37076. 38306554 10.1097/MD.0000000000037076 PMC10843490

[10] Ramaswamy AG Kumaraswamy V Patil N Pattanshetti V. Arthroscopic excision of osteoid osteoma of the femoral neck. Indian J Orthop. 2018;52:568–71. 30237616 10.4103/ortho.IJOrtho_390_17 PMC6142806

[11] Wang F Wang R Zhang C Song E Li F. Clinical effects of arthroscopic-assisted uni-portal spinal surgery and unilateral bi-portal endoscopy on unilateral laminotomy for bilateral decompression in patients with lumbar spinal stenosis: a retrospective cohort study. J Orthop Surg Res. 2024;19:167. 38444008 10.1186/s13018-024-04621-2 PMC10916320

[12] Efthymiadis A Tsikopoulos K Uddin F. Which is the optimal minimally invasive treatment for osteoid osteoma of the hip? A systematic review and proportional meta-analysis. J Orthop Sci. 2022;27:456–62. 33563522 10.1016/j.jos.2020.12.026

